# Dynamic Splitting Performance and Energy Dissipation of Fiber-Reinforced Concrete under Impact Loading

**DOI:** 10.3390/ma17020421

**Published:** 2024-01-14

**Authors:** Dashun Cui, Limin Wang, Chunwei Zhang, Huiting Xue, Dianwei Gao, Fanxiu Chen

**Affiliations:** 1 School of Civil Engineering, Qingdao University of Technology, Qingdao 266033, China; 2 School of Science, Qingdao University of Technology, Qingdao 266033, China; wanglimin@qut.edu.cn (L.W.);; 3 China Construction Second Engineering Bureau LTD. East China Company, Shanghai 200120, China; 4 School of Architecture and Civil Engineering, Shenyang University of Technology, Shenyang 110870, China

**Keywords:** fiber-reinforced concrete, split Hopkinson pressure bar (SHPB) device, dynamic splitting test, energy dissipation

## Abstract

In this paper, the influence of different fiber materials on the dynamic splitting mechanical properties of concrete was investigated. Brazil disc dynamic splitting tests were conducted on plain concrete, palm fiber-reinforced concrete, and steel fiber-reinforced concrete specimens using a split Hopkinson pressure bar (SHPB) test device with a 100 mm diameter and a V2512 high-speed digital camera. The Digital Image Correlation (DIC) technique was used to analyze the fracture process and crack propagation behavior of different fiber-reinforced concrete specimens and obtain their dynamic tensile properties and energy dissipation. The experimental results indicate that the addition of fibers can enhance the impact toughness of concrete, reduce the occurrence of failure at the loading end of specimens due to stress concentration, delay the time to failure of specimens, and effectively suppress the expansion of cracks. Steel fibers exhibit a better crack-inhibiting effect on concrete compared to palm fibers. The incident energy for the three types of concrete specimens is roughly the same under the same impact pressure. Compared with plain concrete, the energy absorption rate of palm fiber concrete is decreased, while that of steel fiber concrete is increased. Palm fiber-reinforced concrete and steel fiber-reinforced concrete have lower peak strains than plain concrete under the same loading duration. The addition of steel fibers significantly impedes the internal cracking process of concrete specimens, resulting in a relatively slow growth of damage variables.

## 1. Introduction

Concrete is the most widely used building material currently, playing a pivotal role in the field of civil engineering [1]. However, it has certain disadvantages, such as low tensile strength, poor crack resistance, and limited ductility. Adding fibers to concrete to create fiber-reinforced concrete (FRC) is one effective method of enhancing the mechanical properties of concrete. This method uses concrete as the matrix and incorporates various types of reinforcing materials, including metal fibers, inorganic non-metallic fibers, synthetic fibers, or natural organic fibers. It fully leverages the inherent properties of concrete and the complex interactions between fibers and concrete to achieve superior mechanical performance [2,3,4,5,6].

The research on fiber-reinforced concrete began in the early 20th century, with steel fiber-reinforced concrete developing the fastest. Steel fiber-reinforced concrete has been widely used in various fields, including bridges, tunnel linings, road surfaces, and precast components [7]. Previous studies have shown that, during the concrete curing process, steel fibers can effectively reduce concrete shrinkage and the number of cracks, while enhancing strength and ductility [8,9]. Song et al. [10] studied the mechanical properties of high-strength steel fiber-reinforced concrete and found that the compressive strength, tensile strength, and toughness index of steel fiber-reinforced concrete all improved with the volume fraction of steel fibers increased. Due to the obvious strain rate effect and the discrete nature of concrete materials, the influence of fibers on the dynamic mechanical properties of concrete is much more complicated than on static properties [11,12]. Park et al. [13] researched the direct tensile stress and strain response of ultra-high-performance steel fiber-reinforced concrete under different strain rates and found that the tensile strength of specimens significantly increased at high strain rates compared to static strain rates. Additionally, Ulzurrun et al. [14] investigated the influence of steel fibers on the impact resistance of reinforced concrete beams and found that the addition of steel fibers can enhance the impact strength and ductility of reinforced concrete beams, reduce brittleness, and improve their plasticity characteristics. The failure mode shifted from brittle shear failure without fibers to flexural failure with fibers. Although impact caused a mesh of cracks, the crack-bridging effect of steel fibers prevented crack propagation, significantly improving the impact resistance of the concrete beams. Ye et al. [15] conducted dynamic impact compression tests on steel fiber-reinforced concrete specimens and observed that the peak strain and peak stress of the steel fiber-reinforced concrete specimens increased with a higher fiber content and strain rate.

Some plant fibers have been receiving increased attention from scientists in recent years as low-carbon environments have become increasingly important. A study conducted by Kumar et al. [16] using coconut fiber and coconut fiber ash in concrete found that 5% coconut fiber and 15% coconut fiber ash could improve the durability of concrete. MD et al. [17] explored the mechanical properties and impact resistance of concrete reinforced with coconut fiber (CF) and polypropylene fiber (PPF). The greatest increase in strength was observed when the total fiber content of the hybrid fiber concrete was 0.3%, and the increase in impact resistance of the hybrid fiber concrete was almost twice that of the individual fiber concrete and three times that of the plain concrete. In recent years, the application of synthetic organic fibers, represented by polypropylene fiber (PPF), has made great progress. Song et al. [18] conducted an experimental study on the mechanical and flexural properties of polypropylene fiber-reinforced concrete, and the results showed that the fibers can inhibit crack expansion and avoid brittle damage when cracks appear in the concrete. Yao et al. [19] investigated the mechanical properties of polypropylene fiber-reinforced concrete, and the results showed that the flexural strength, tensile strength, and fracture toughness of polypropylene fiber-reinforced concrete were significantly higher than those of ordinary concrete. Afroughsabet et al. [20] investigated the effect of adding steel and polypropylene fibers on the mechanical properties of high-strength concrete, and the results showed that the addition of both steel and polypropylene fibers significantly increased the split tensile and flexural strengths of concrete.

Current research primarily focuses on the influence of fiber addition on the mechanical properties of concrete, with limited attention given to the damage evolution characteristics during the failure process of fiber-reinforced concrete and the fracture toughness after failure. Furthermore, there is relatively little comparative research on the mechanical properties of different types of fiber-reinforced concrete. Consequently, this article uses a high-speed digital camera and a split Hopkinson pressure bar (SHPB) test device to conduct dynamic splitting tests on plain concrete, palm fiber-reinforced concrete, and steel fiber-reinforced concrete specimens. The mechanical parameters, such as dynamic tensile properties and the damage evolution of different fiber concrete specimens during impact damage, have been thoroughly analyzed and discussed, and the dynamic crack propagation and strain field variation of the specimen surfaces were analyzed with image processing technology, which can provide important references and guides for the design of impact resistance of concrete structures.

## 2. Experimental Methodology

### 2.1. Preparation of Specimens

The specimens were prepared using ordinary Portland cement with a strength grade of 42.5 (Zhangqiu Huaming Cement Co., Ltd., Jinan, China) as the binder material. The coarse aggregate consisted of crushed stones with particle sizes less than 10 mm, while the fine aggregate was river sand (Zhangqiu Shuangshan sand and stone factory, Jinan, China). The proportion of the mixture is shown in Table 1.

The fiber materials included common palm fibers and hooked-end steel fibers (Laiwu Xingtai Engineering Materials Co., Ltd., Jinan, China), with the main parameters of the fiber materials shown in Table 2. Three types of specimens were prepared: plain concrete specimens (PC), palm fiber-reinforced concrete specimens (PFRC), and steel fiber-reinforced concrete specimens (SFRC). All specimens were cylindrical, with a diameter of 120 mm and a height of 100 mm.

To evaluate the basic tensile mechanical properties of the specimens, a quasi-static Brazilian disc splitting test was conducted. The tensile strengths of plain concrete, palm fiber-reinforced concrete, and steel fiber-reinforced concrete were 3.7 MPa, 3.7 MPa, and 4.2 MPa, respectively.

### 2.2. SHPB Experimental Device

The split Hopkinson pressure bar (SHPB) test device is based on two fundamental assumptions: the one-dimensional elastic stress wave assumption and the assumption of stress uniformity. It utilizes stress waves generated during high-speed impact on a specimen by elastic bars to study the dynamic mechanical properties of materials [21]. Using the SHPB device as a loading tool for the dynamic splitting test, when the impact bar impacts the incident bar from the launcher chamber, an incident pulse will be generated in the incident bar. This incident pulse propagates continuously along the incident bar, through the specimen, and into the transmission bar. Due to the different wave impedances between the specimen and the pressure bars, the incident pulse undergoes reflection and transmission at the interface between the specimen and the pressure bars. A portion of it reflects back into the incident bar, forming a reflected wave, while the other part continues to propagate forward, inducing high-speed deformation in the specimen. The pulse that passes through the specimen enters the transmission bar, forming a transmitted wave. The incident wave, reflected wave, and transmitted wave are collected through strain gauges attached to the pressure bars. The axial load borne by the specimen is calculated as shown in the formula [22]:

(1) $$P\left(t\right)=E_{0}A_{0}\left[\varepsilon_{i}(t)-\varepsilon_{r}(t)\right]=E_{0}A_{0}\varepsilon_{t}(t)$$

where $E_{0}$ represents the elastic modulus of the pressure bar, $A_{0}$ is the cross-sectional area of the pressure bar, $\varepsilon_{i}$, $\varepsilon_{r}$, and $\varepsilon_{t}$, respectively, represent the strains of the incident wave, reflected wave, and transmitted wave.

**Figure 1:**
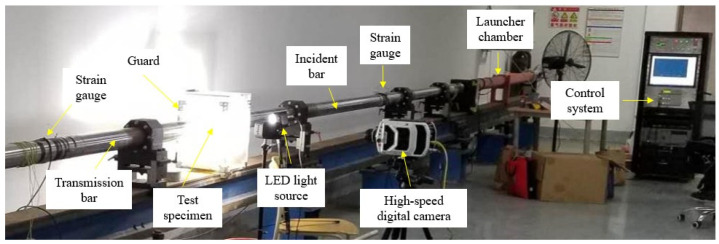
Split Hopkinson pressure bar (SHPB) test device.

The SHPB test device (Changsha Baisen experimental equipment Co., Ltd., Changsha, China) used in this study is shown in Figure 1. The diameter of the pressure bars is 100 mm, the elastic modulus is 210 GPa, and the density is 7850 kg/m^3^. The lengths of the incident bar, transmitted bar, and impact bar are 6 m, 4 m, and 2 m.

### 2.3. Brazilian Disc Splitting Test

**Figure 2:**
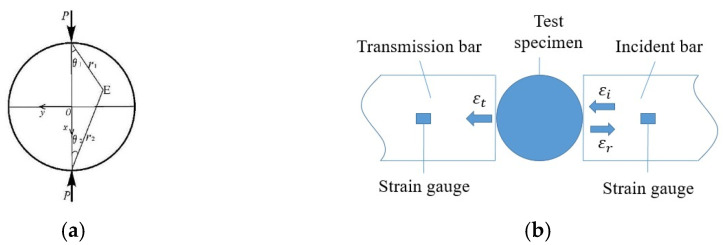
Schematic diagram of Brazilian disc splitting test, (**a**) Brazilian disc force model; (**b**) SHPB split tensile test.

Indirect tensile methods are commonly used to obtain the tensile strength of brittle materials. Among these methods, the Brazilian disc splitting test is one of the most widely applied measurement techniques. The Brazilian disc splitting test was developed based on the theory of two-dimensional plane elasticity. Its theoretical model involves a circular specimen subjected to a pair of equivalent reverse radial forces, as shown in Figure 2a. Figure 2b is the clamping position of the specimen in the SHPB test device.

Concrete has significantly lower tensile strength compared to its compressive strength. Loading the concrete in the manner shown in Figure 2b will result in brittle tensile failure, with the maximum tensile stress uniformly distributed along the line connecting the radial forces at both ends of the disc. The calculation formula for the dynamic tensile stress of the specimen is [23]:

(2) $$\sigma_{dt}\left(t\right)=\frac{2P\left(t\right)}{\pi DH}=\frac{2E_{0}A_{0}\varepsilon_{t}(t)}{\pi DH}$$

where $\sigma_{dt}\left(t\right)$ represents the dynamic tensile stress of the specimen, $P\left(t\right)$ stands for the axial load applied to the specimen, D is the diameter of the specimen’s circular cross-section, and H is the height or thickness of the specimen.

When the specimen undergoes fracture failure, the transmitted waves will reach their stress peak, and the corresponding maximum tensile stress at this moment represents the dynamic tensile strength of the specimen.

### 2.4. SHPB Test Energy Dissipation

When using the SHPB test device to conduct the dynamic Brazilian disc splitting test, the high-pressure gas energy is first converted into incident energy. When the incident energy acts on the specimen, a portion of it is converted into reflected energy, while another portion is absorbed by the energy-absorbing device through the transmission bar. The remaining portion is primarily absorbed by the specimen, causing it to fracture and dissipate energy in the process. According to the principle of energy conservation, the energy absorbed by the specimen during the dynamic splitting test of SHPB is [24]:

(3) $$W_{s}\left(t\right)=W_{i}\left(t\right)-W_{r}\left(t\right)-W_{t}\left(t\right)$$

where $W_{i}\left(t\right)$, $W_{r}\left(t\right)$, and $W_{t}\left(t\right)$ represent the energy carried by the incident wave, reflected wave, and transmitted wave, which can be obtained with the following formula:

(4) $$W_{i}\left(t\right)=E_{0}C_{0}A_{0}\int_{0}^{t}{\varepsilon_{i}}^{2}\left(t\right)dt$$

(5) $$W_{r}\left(t\right)=E_{0}C_{0}A_{0}\int_{0}^{t}{\varepsilon_{r}}^{2}\left(t\right)dt$$

(6) $$W_{t}\left(t\right)=E_{0}C_{0}A_{0}\int_{0}^{t}{\varepsilon_{t}}^{2}\left(t\right)dt$$

where $E_{0}$ represents the elastic modulus of the pressure bar, $C_{0}$ stands for the longitudinal wave velocity of the pressure bar, $A_{0}$ denotes the cross-sectional area of the pressure bar, and $\varepsilon_{i}$, $\varepsilon_{r}$, and $\varepsilon_{t}$ correspond to the strains of the incident wave, reflected wave, and transmitted wave on the pressure bar, respectively.

To further analyze the energy absorption characteristics of the specimen, the absorption efficiency is defined as the ratio of the energy absorbed by the specimen to the incident energy.

(7) $$\eta =\frac{W_{s}}{W_{i}}$$

The absorption efficiency is a measure of how effectively the specimen absorbs energy from the incident wave, providing insight into its energy dissipation properties during dynamic tests.

### 2.5. High-Speed Camera and DIC Technology

High-speed camera technology provides the capability to capture the expansion and trends of concrete cracks during dynamic loading processes with great clarity. When employing the V2512 ultra-high-speed digital camera (Vision Resesarch Inc., Wayne, NJ, USA) for the dynamic splitting test, a shooting configuration of 512 × 320 @ 110,000 fps was selected. This means that the resolution of the captured frames is 512 × 320, with a frame rate of 110,000 frames per second, and a time interval of 9.08 μs between each frame. The actual size of a unit pixel in the captured frames is 0.443 mm after fixing the camera’s position and shooting angle.

Digital Image Correlation (DIC) is a measurement technique used in the field of optics [25]. It is widely employed in various engineering disciplines. It leverages the movement of speckles on the surface of a deforming object to calculate parameters such as full-field strain and full-field displacement. DIC accurately observes and computes the displacement changes of these speckles on the specimen’s surface, providing valuable insights into the object’s behavior.

## 3. Experimental Results and Analysis

### 3.1. Impact Failure Morphology

**Figure 3:**
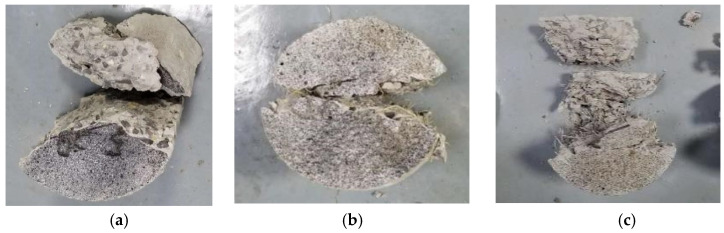
Impact failure morphology of specimens: (**a**) plain concrete; (**b**) palm fiber-reinforced concrete; (**c**) steel fiber-reinforced concrete.

The impact failure morphology of the specimens is shown in Figure 3. The main damage area of the specimen includes the contact region between the specimen and the pressure bar, as well as the geometric center of the specimen. This observation aligns with the expectations of the Brazilian disc splitting test theory. The contact area between the specimen and the pressure bar is relatively small, resulting in significant stress concentration after loading and leading to partial compression failure in that region. The incident stress wave passes through the contact surface between the pressure bar and the specimen, propagating into the interior of the specimen. The geometric center of the circular specimen is subjected to tensile stress. When the specimen reaches its tensile strength limit, tensile failure extends radially towards both ends of the specimen. As the crack width increases, the specimen is gradually split along the radial direction into two approximately semicircular pieces.

The fracture surfaces of plain concrete and palm fiber-reinforced concrete are relatively smooth, consistent with brittle fracture characteristics. In contrast, the fracture surface of steel fiber-reinforced concrete is more fragmented, with numerous straightened steel fibers and concrete fragments adhered to the interface. This indicates that, after reaching their tensile strength in the central part of the specimen and undergoing fracture failure, the steel fibers played a bridging role on the fracture surface. These steel fibers were tightly bonded to the concrete matrix and disrupted the surface of the matrix during the pull-out process. From the perspective of fracture morphology, the bridging effect of palm fibers on the fracture surface is not very pronounced. Some palm fibers are partially torn, and some are pulled out. This is related to the lower strength of palm fibers.

### 3.2. Crack Dynamic Propagation

**Figure 4:**
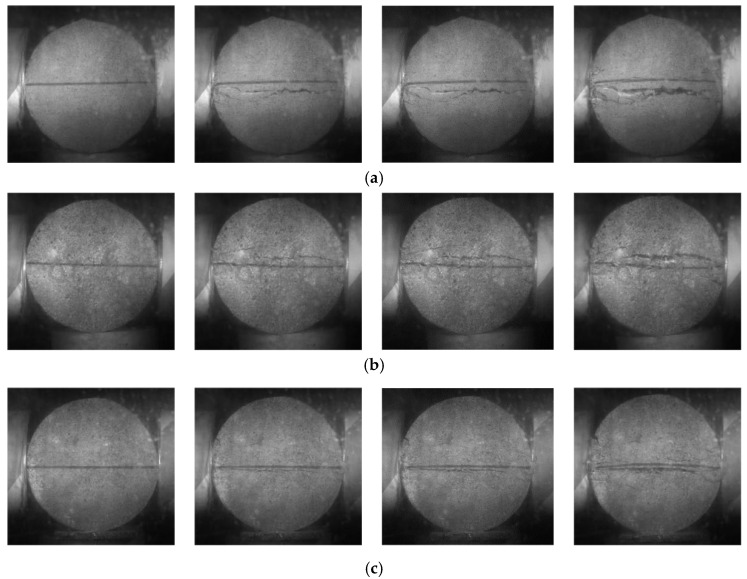
Comparison diagram of crack dynamic propagation of three types of concrete specimens at 0 μs, 454 μs, 681 μs, and 908 μs. (**a**) Plain concrete; (**b**) palm fiber-reinforced concrete; (**c**) steel fiber-reinforced concrete.

Solely observing the ultimate failure morphology of the specimens is insufficient for understanding the contributions of fibers, and it may not effectively illustrate the bridging role of fibers during the crack propagation process. By extracting images of crack propagation captured by the high-speed camera, we can observe the entire dynamic process of crack propagation and make lateral comparisons of crack width changes at various time intervals for different specimens, as shown in Figure 4. The left and right ends of the Brazilian disc specimens correspond to the transmitting bar and the incident bar faces, respectively.

The fracture of the specimen is initiated from the center point at the contact area of the pressure bar and propagates along the loading diameter direction, which satisfies the validity conditions of the Brazilian disc splitting test. Based on the comparison of crack dynamic propagation images of the specimens, it is evident that the inclusion of fibers can significantly hinder the occurrence and development of macroscopic cracks, and steel fibers have a more effective role than palm fibers in this regard. At 454 μs, it is clearly visible that the crack in the plain concrete specimen has completely penetrated radially, while the palm fiber-reinforced concrete and steel fiber-reinforced concrete specimens only exhibit faint crack zones. By 681 μs, the plain concrete specimen had completely fractured along its centerline, with the upper and lower blocks showing an accelerating separation trend. At this point, the crack in the palm fiber-reinforced concrete specimen has just penetrated and slightly opened, while the crack in the steel fiber-reinforced concrete specimen has only penetrated the specimen without any sign of widening. At 908 μs, the crack width in the plain concrete specimen continues to increase, and there is also noticeable expansion in the crack of the palm fiber-reinforced concrete specimen. The crack width in the steel fiber-reinforced concrete specimen is significantly smaller than that in the plain concrete and palm fiber-reinforced concrete specimens.

Palm fibers and steel fibers have similar effects during the initial stage of impact loading; they can delay the occurrence and penetration time of cracks. However, after the cracks have completely propagated, the bridging effect of palm fibers quickly fails as they are either pulled apart or elongated along with the crack opening. In contrast, steel fibers can still bond the crack surfaces and delay crack propagation.

### 3.3. Crack Width Propagation

**Figure 5:**
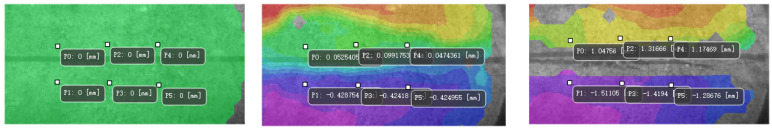
Schematic diagram of crack width calculation.

**Figure 6:**
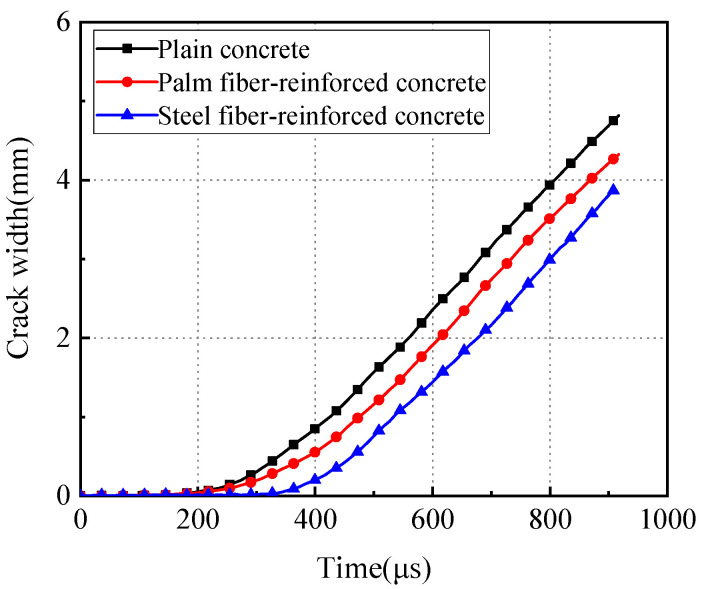
Crack width variation diagram.

The Digital Image Correlation software Vic-2D version 6.2.0 (Correlated Solutions Inc., Columbia, SC, USA) was used to process the speckle images captured during the impact failure process of the specimens, as shown in Figure 5. Initially, three sets of measurement points corresponding to upper and lower positions were selected on the speckle image of the specimen. Then, the software was employed to measure the crack width between these three sets of measurement points, and the arithmetic average of the crack widths from the three sets was calculated as the final crack propagation width. Finally, the variation in crack width on the specimen’s surface over time was obtained, as illustrated in Figure 6.

It can be observed that, under the impact load, plain concrete is the first to develop cracks, followed by palm fiber-reinforced concrete, while the cracking initiation of steel fiber-reinforced concrete noticeably lags behind the former two types. Furthermore, at the same time point, the crack widths of palm fiber-reinforced concrete and steel fiber-reinforced concrete were significantly smaller than those of plain concrete, indicating that the inclusion of fibers effectively impedes crack propagation. Moreover, steel fibers exhibit a better crack resistance effect on concrete compared to palm fibers.

### 3.4. Dynamic Tensile Stress Analysis

**Figure 7:**
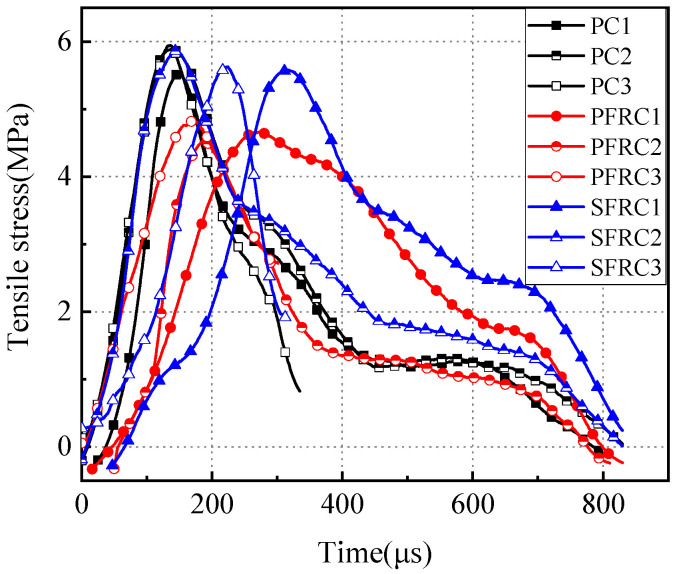
Dynamic tensile stress time–history curve of the specimens. Plain concrete specimens (PC), palm fiber-reinforced concrete specimens (PFRC), and steel fiber-reinforced concrete specimens (SFRC).

Through the processing of pulse data recorded by the strain gauges on the incident and transmission bars, in combination with the parameters of the specimen and pressure bar, the dynamic tensile stress curve of the specimens with time can be obtained, as shown in Figure 7.

For concrete-like materials, it is common to use a dynamic increase factor (DIF) to describe their dynamic mechanical properties. This factor is defined as the ratio of the specimen’s dynamic strength to its quasi-static strength. The calculated results are presented in Table 3.

It can be observed in Table 3 that palm fiber-reinforced concrete and steel fiber-reinforced concrete have a longer time to impact failure compared to plain concrete under the same impact load. The addition of the two fibers enhances the impact resistance of the concrete material.

The dynamic increase factors for the splitting tensile strength of plain concrete, palm fiber-reinforced concrete, and steel fiber-reinforced concrete are 1.56, 1.26, and 1.35, respectively. These values indicate that the dynamic tensile strength is higher than the quasi-static tensile strength, demonstrating a significant strain rate effect.

### 3.5. Energy Dissipation Analysis

**Figure 8:**
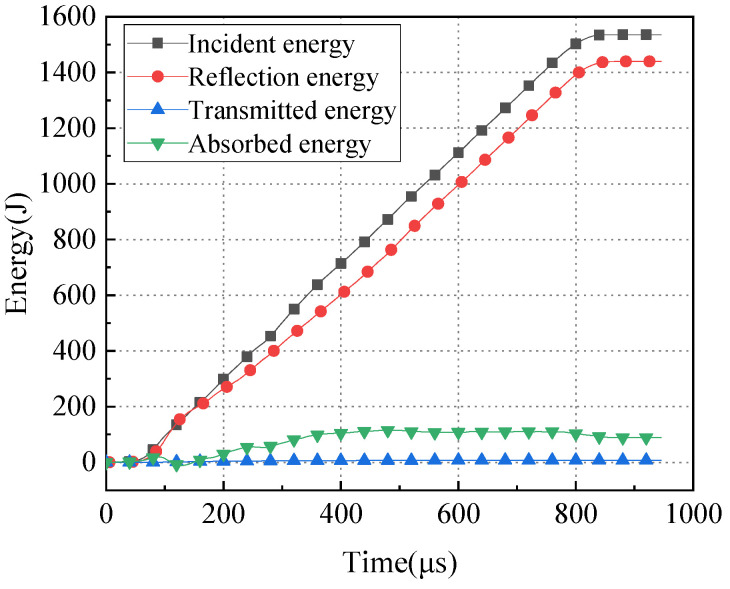
Time–history curve of energy change in steel fiber-reinforced concrete.

Based on the strain measurements of incident waves, reflected waves, and transmitted waves in the split Hopkinson pressure bar test, the energy carried by these waves can be calculated. Taking a steel fiber-reinforced concrete specimen as an example, the energy variation over time during the dynamic splitting process of the specimen is shown in Figure 8.

In the initial stage of the SHPB dynamic tensile test, the incident energy, reflected energy, and absorbed energy of the specimen all increase with time until they reach a certain point where the energy levels remain relatively constant, indicating that energy is no longer increasing. In contrast, the transmitted energy shows minimal variation with time, and its energy change curve is approximately horizontal.

It may be seen in Figure 8 that the reflected energy is relatively large, while the transmitted energy is very small and significantly less than the reflected energy. This is because the contact area between the specimen and pressure bar is quite small. When the incident energy reaches the end of the incident bar, a significant portion of it returns into the incident bar as reflected energy, and the remaining energy is absorbed by the specimen, forming absorbed energy. Only a small fraction of the energy passes through the disc specimen and forms transmitted energy in the transmitted bar. The energy data for the specimens during the dynamic splitting process is presented in Table 4.

The magnitude of the impact air pressure determines the size of the incident energy. Under the same impact air pressure, the incident energy for the three types of concrete specimens is roughly the same. Palm fiber-reinforced concrete has a slightly reduced energy absorption rate compared to plain concrete, while steel fiber-reinforced concrete specimens have an increased energy absorption rate.

### 3.6. Dynamic Strain Analysis

**Figure 9:**
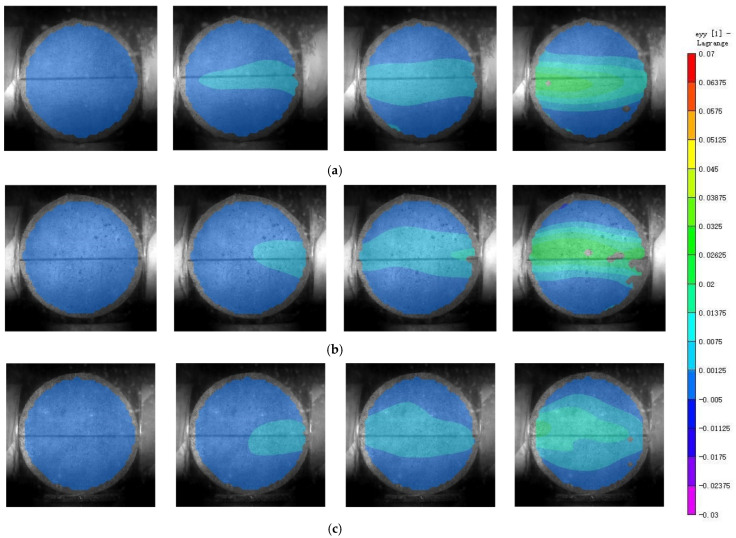
Strain cloud diagram of the specimens at 0 μs, 181.6 μs, 272.4 μs, and 363.2 μs. (**a**) Plain concrete; (**b**) palm fiber-reinforced concrete; (**c**) steel fiber-reinforced concrete.

Using the software Vic-2D to process speckle images of the specimen captured by high-speed camera, a strain cloud diagram of the specimen surface changing with time under the impact load was obtained, as shown in Figure 9.

From the strain cloud diagram of the specimens, the strain evolution of the three concrete specimens during the dynamic splitting process can be clearly seen. At 181.6 μs, the internal strain distribution in all three concrete specimens is not stable, with the strain primarily concentrated in the area in contact with the incident bar. Subsequently, the strain propagates inward along the radial direction of the specimen. By 272.4 μs, the internal strain in the concrete specimens becomes more uniform after multiple reflections of the stress waves. Based on the strain cloud diagram, it can be observed that the strain is mainly distributed along the line connecting the end points of the specimen. The maximum strain in the specimen occurs at this location and decreases outward from this central point. When the time reaches 363.2 μs, the strain in the specimen further increases. At this time, the maximum strain values for plain concrete and palm fiber-reinforced concrete are quite similar, while the maximum strain value for steel fiber-reinforced concrete is noticeably smaller than the former two.

### 3.7. Damage Variable Analysis

Concrete specimens may generate some initial cracks within them during the forming process. These initial cracks continue to propagate and connect due to the action of loads, and new cracks will be generated. This leads to the deterioration of the macroscopic mechanical properties of the concrete specimen. Therefore, the damage evolution in concrete can be seen as the formation and propagation of various microcracks. Damage mechanics introduces the concept of damage variables, which are physical quantities reflecting the internal defect state of a material. For concrete materials, damage variables can be determined by measuring changes in the material’s elastic modulus. Damage variables are defined using Young’s modulus and can be represented as follows [26]:

(8) $$D=1-\frac{\tilde{A}}{A}=1-\frac{\tilde{E}}{E}$$

where E represents the initial Young’s modulus, determined by the initial slope of the stress–strain curve, and $\tilde{E}$ represents the effective Young’s modulus, determined by the tangent slope of the stress–strain curve.

**Figure 10:**
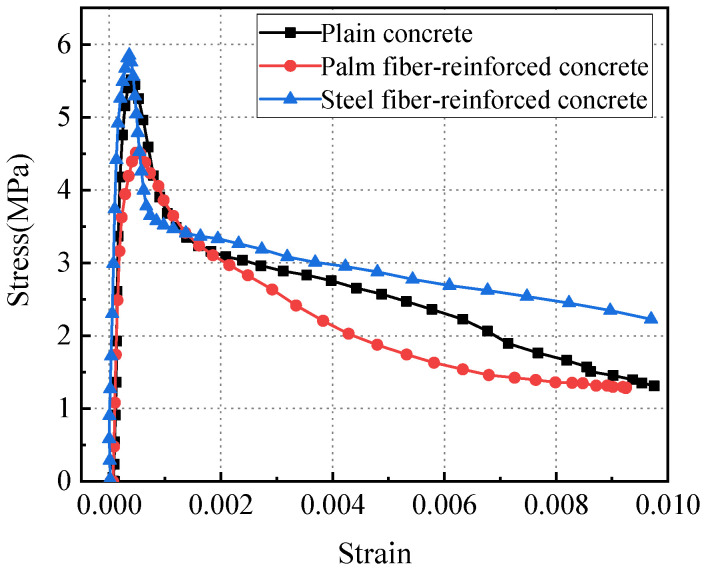
Dynamic splitting stress–strain curve.

According to the Brazilian disc splitting tensile stress and the strain obtained through DIC software Vic-2D processing, the dynamic tensile stress–strain curves for the three types of concrete specimens under impact loads are plotted as shown in Figure 10.

From Figure 10, it is evident that the initial rising trend of the dynamic tensile stress–strain curves for the three types of concrete specimens is essentially the same. However, after reaching the peak stress, there is a sharp drop in the stress–strain curve for all three types of concrete, but the shapes of the descending segment are different. Cracks in steel fiber-reinforced concrete expand due to instability, and their load-bearing capacity decreases. However, due to the bridging effect of steel fibers in cracks, the bearing capacity of the concrete does not completely disappear. The descending segment of the stress–strain curve for steel fiber-reinforced concrete is gentler than plain concrete and palm fiber-reinforced concrete. The addition of steel fibers improves the fracture toughness of concrete.

**Figure 11:**
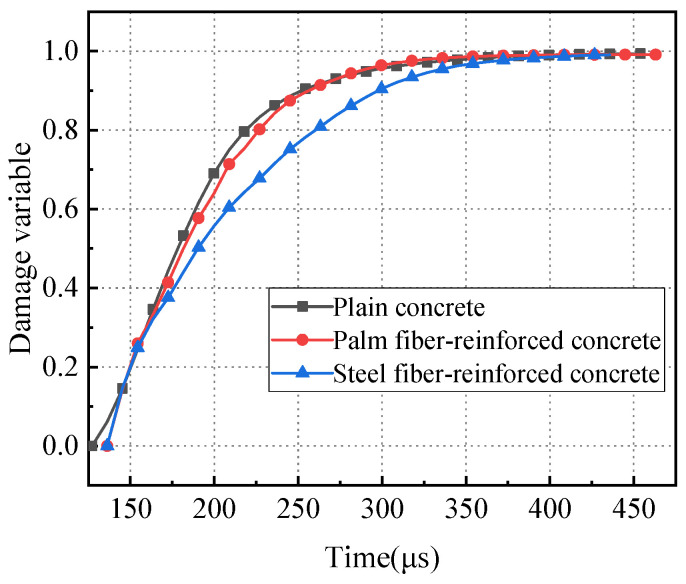
Change curve of damage variables.

The initial Young’s modulus of the three kinds of concrete specimens was calculated by taking 60% peak stress and the corresponding strain as the representative values in this paper. The initial Young’s modulus of plain concrete, palm fiber concrete, and steel fiber concrete are 21.19 GPa, 16.03 GPa, and 25.99 GPa, respectively. Based on the dynamic tensile stress–strain curves for the three types of concrete specimens and Equation (8), the relationship between the specimen damage variables and the time of impact loading was determined, as shown in Figure 11.

It may be seen in Figure 11 that the trends of damage variable curves for the three types of concrete specimens are similar and can be roughly divided into two stages. In the first stage, which occurs approximately before 200 μs, the damage variables for the specimens develop rapidly, reflecting the rapid growth of internal defects within the specimens. The maximum damage variables for plain concrete, palm fiber-reinforced concrete, and steel fiber-reinforced concrete are 0.69, 0.64, and 0.56, respectively. Notably, steel fiber-reinforced concrete has the smallest damage variables. In the second stage, the rate of growth of the damage variables for the specimens slows down, indicating slower development of internal defects within the specimens until the specimens eventually fail. During this stage, the trends in damage variable growth for plain concrete and palm fiber-reinforced concrete are very similar, while the damage variable for steel fiber-reinforced concrete is noticeably smaller than the other two. This suggests that the inclusion of steel fibers can impede the development of internal defects within concrete specimens and enhance the impact resistance of concrete.

## 4. Conclusions

Dynamic Brazilian disc splitting tests were conducted on plain concrete, palm fiber-reinforced concrete, and steel fiber-reinforced concrete specimens using a split Hopkinson pressure bar (SHPB) test device. Combining high-speed photography and Digital Image Correlation (DIC) technology, this paper analyzed dynamic mechanical properties such as impact failure morphology, crack dynamic expansion, dynamic tensile stress, energy dissipation, dynamic strain, and the damage variables of concrete specimens under impact loading, researched the influence of palm fiber and steel fiber on the dynamic mechanical properties of concrete, and derived the following conclusions:

(1) The inclusion of fibers can enhance the impact toughness of concrete, better transmit the impact load, and reduce the occurrence of failure at the loading end of the specimens due to stress concentration. Furthermore, fibers play a significant role in impeding the propagation of macrocracks within the specimens. Notably, steel fibers exhibit a more prolonged action duration compared to palm fibers. In dynamic splitting tests, the failure mode observed for steel fibers is fiber pull-out, while palm fibers tend to fail through fiber rupture.
(2) The dynamic tensile strength of plain concrete, palm fiber-reinforced concrete, and steel fiber-reinforced concrete specimens surpasses their static tensile strength. Plain concrete exhibits the highest dynamic increase factor, followed by steel fiber-reinforced concrete, and palm fiber-reinforced concrete has the lowest enhancement.
(3) The incident energy for the three types of concrete specimens remains largely consistent at the same impact pressure. Compared to plain concrete, palm fiber-reinforced concrete shows a slightly reduced energy absorption rate, while steel fiber-reinforced concrete specimens exhibit an increased energy absorption rate.
(4) Palm fiber-reinforced concrete and steel fiber-reinforced concrete exhibit lower peak strains compared to plain concrete at the same time of impact loading. The inclusion of steel fibers significantly impedes the internal fracture process of the concrete specimens, resulting in a relatively slower growth of damage variables, thereby enhancing their impact resistance and durability.

Overall, compared with plain concrete and palm fiber-reinforced concrete, steel fiber-reinforced concrete shows better impact resistance under impact loading, which can provide an important reference and guidance for the design of impact resistance in concrete structures.

## Figures and Tables

**Table 1:** Mix proportions of concrete specimens (kg/m^3^).

Concrete Types	Water	Cement	Sand	Aggregate	Fly Ash	Water Reducer	Palm Fiber	Steel Fiber
Plain concrete	206	378	723	891	47	4.58	0	0
Palm fiber-reinforced concrete	206	378	723	891	47	4.58	10	0
Steel fiber-reinforced concrete	206	378	723	891	47	4.58	0	156

**Table 2:** Main parameters of fiber materials.

Fiber Types	Length (mm)	Diameter (mm)	Length–Diameter Ratio	Tensile Strength (MPa)	Density (kg/m^3^)
Palm fiber	35	0.38	92	150	500
Steel fiber	35	0.54	65	1300	7800

**Table 3:** Dynamic tensile strength of specimens.

Material Type	Specimen Number	Failure Time (μs)	Average Failure Time (μs)	Dynamic Tensile Strength (MPa)	Average Dynamic Tensile Strength (MPa)	Static Tensile Strength (MPa)	Dynamic Increase Factor
Plain concrete	PC1	148.40	140.40	5.52	5.78	3.70	1.56
	PC2	137.20		5.88			
	PC3	135.60		5.94			
Palm fiber-reinforced concrete	PFRC1	273.20	209.87	4.66	4.67	3.70	1.26
	PFRC2	186.40		4.53			
	PFRC3	170.00		4.83			
Steel fiber-reinforced concrete	SFRC1	318.00	228.53	5.58	5.69	4.20	1.35
	SFRC2	143.60		5.86			
	SFRC3	224.00		5.63			

**Table 4:** Energy dissipation analysis of specimens.

Material Type	Specimen Number	Incident Energy (J)	Reflected Energy (J)	Transmitted Energy (J)	Absorbed Energy (J)	Energy Absorption Rate (%)	Average Energy Absorption Rate (%)
Plain concrete	PC1	1641.15	1579.57	8.35	53.23	3.24	4.00
	PC2	1586.66	1497.49	6.86	82.31	5.19	
	PC3	1687.72	1619.57	7.92	60.23	3.57	
Palm fiber-reinforced concrete	PFRC1	1564.10	1511.99	7.24	44.87	2.87	2.94
	PFRC2	1556.35	1506.43	3.56	46.36	2.98	
	PFRC3	1579.66	1525.69	6.83	47.14	2.98	
Steel fiber-reinforced concrete	SFRC1	1613.68	1522.63	8.31	82.74	5.13	5.40
	SFRC2	1535.73	1439.63	7.45	88.65	5.77	
	SFRC3	1590.82	1499.42	7.09	84.31	5.30	

## Data Availability

Data available upon reasonable request to corresponding author.
